# Evaluation of radiation damage effects of low-energy Auger and conversion electrons emitted by ^134^Ce decay using a Monte Carlo method

**DOI:** 10.1038/s41598-026-58223-5

**Published:** 2026-06-21

**Authors:** Sangho Lee, Wonku Kim, Deokseong Kim, Jungwook Shin, Yoonsuk Huh, Kondapa Naidu Bobba, Robert R. Flavell, Gyuseong Cho, Youngho Seo

**Affiliations:** 1 Department of Nuclear and Quantum Engineering, Korea Advanced Institute of Science and Technology, Daejeon, 34141 Republic of Korea; 2 Department of Radiology and Biomedical Imaging, University of California, San Francisco, CA 94107 USA; 3 Radioisotope Research Division, Korea Atomic Energy Research Institute, Daejeon, 34057 Republic of Korea; 4 Division of Cancer Epidemiology and Genetics, National Cancer Institute, National Institutes of Health, Rockville, MD 20850 USA; 5 Department of Radiation Oncology, Seoul National University Hospital, Seoul, 03080 Republic of Korea; 6 Institute of Radiation Medicine, Seoul National University Medical Research Center, Seoul, 03080 Republic of Korea; 7 Department of Nuclear Engineering, University of Tennessee, Knoxville, TN 37996 USA; 8 Department of Pharmaceutical Chemistry, University of California, San Francisco, CA 94158-2517 USA; 9 Department of Nuclear Engineering, University of California, Berkeley, CA 94720 USA

**Keywords:** ^134^Ce, Auger electrons, Internal conversion electrons, Targeted radionuclide therapy, Monte Carlo simulation, TOPAS-nBio, Biophysics, Cancer, Oncology, Physics

## Abstract

Targeted radionuclide therapy (TRT) is a cancer treatment method that delivers radiation to specific tumor cells, enabling efficient tumor cell killing. Radionuclides emitting short-range beta or alpha particles have previously been the primary focus. TRT approaches utilizing low-energy electrons, such as Auger and internal conversion electrons, have attracted interest because of their highly localized tumor-killing potential. ^134^Ce is an imaging surrogate in the form of a ^134^Ce/^134^La pair for positron emission tomography (PET) imaging in ^225^Ac targeted alpha therapy, and it has recently exhibited promising therapeutic properties. This study employs TOPAS-nBio, a Monte Carlo simulation tool, to investigate the radiation damage effects of ^134^Ce from dosimetric and DNA-scale perspectives. The DNA damage yield analysis incorporates both physical and chemical processes. In the water sphere geometry, the overall dose contribution of ^134^Ce shows patterns generally similar to those of other Auger-emitting radionuclides, and ^134^Ce shows damage yields per decay close to those of ^161^Tb on the DNA scale. Although ^134^Ce induces fewer DNA double-strand breaks per decay in the nucleus than ^125^I and ^161^Tb, it exhibits a higher double-strand break yield when normalized to absorbed dose. Such damage outcome predictions suggest that further research on radionuclide therapy using ^134^Ce is worthwhile.

## Introduction

Targeted radionuclide therapy (TRT) is a therapeutic approach in which radionuclides are linked to biomolecules that target specific cancer cells^1^. This strategy allows for more efficient delivery of radiation doses than conventional external beam radiotherapy. Radionuclides emitting high linear energy transfer (LET) particles are typically used, with the choice of isotope depending on factors such as tumor size, pharmacokinetics of the targeting radiopharmaceutical, and biological characteristics. Theoretically, while alpha-emitting radionuclides such as ^225^Ac and ^227^Th are effective in the treatment of metastatic tumors, beta-emitting isotopes such as ^177^Lu are primarily adopted for larger tumors^2^.

Radionuclides emitting low-energy electrons, such as Auger electrons (AEs) and internal conversion electrons (CEs), could be considered promising for micro- and metastatic tumors because of their shorter range compared to alpha and beta particles. While studies on TRT using beta- or alpha-emitting radionuclides have been extensive, those involving low-energy electron-emitting radionuclides have been relatively limited because of their physical properties. Low-energy electrons typically exhibit a lower LET than alpha particles, which are in the MeV range, but a higher LET than conventional beta particles, with reported values in the range of 4–26 keV/µm^3^.

^134^Ce is a low-energy electron-emitting radionuclide that has recently gained attention as a compatible imaging surrogate for alpha-emitting ^225^Ac^4,5^. ^225^Ac is one of the most prominent radionuclides used in targeted alpha therapy^6^, with remarkable therapeutic efficacy in various cancers, including prostate cancer^7^. ^134^Ce has a half-life of 3.16 days and undergoes 100% electron capture, decaying to ^134^La^8^. The daughter nuclide ^134^La decays to stable ^134^Ba with a half-life of 6.45 min and a β^+^ branching ratio of approximately 64%, during which the emitted positrons enable positron emission tomography (PET) imaging^4^. During the decay of ^134^Ce to ^134^La, a considerable number of low-energy electrons are released; however, these have received relatively less attention than the positrons emitted during ^134^La decay^8^.

As ^134^Ce emits low-energy electrons during its decay, it has been proposed as a potential therapeutic isotope for targeted radionuclide therapy and has been evaluated in a prior dosimetric study^8^. However, this study was limited to the estimation of absorbed doses using theoretical models with simplified geometry. Recently, it was reported that ^134^Ce-PSMA-617 demonstrated therapeutic efficacy in preclinical models of prostate cancer^9^. Nevertheless, these studies have not been extended to the analysis of the microdosimetry and damage yield of ^134^Ce at the cellular and DNA levels.

Tumor lethality is often associated with the delivery of a sufficient absorbed dose; however, the induction of DNA double-strand breaks is a more critical determinant of cell death because it causes irreparable damage to tumor cells. In this study, Monte Carlo simulations were performed using the TOPAS-nBio toolkit^10^ to evaluate the microdosimetric characteristics and DNA damage yields of ^134^Ce and to compare them with those of other radionuclides known to emit low-energy electrons.

This investigation examines the potential of ^134^Ce as a candidate for targeted radionuclide therapy. The evaluation was conducted by estimating absorbed doses in a simplified cell geometry and analyzing DNA strand breaks using both a simplified DNA model and a more complex nucleus model. Water was adopted as the primary constituent material for each geometry because of its common use in radiation transport studies. This choice also reflects the high water content of solid tumors, which are the primary targets in most cancer treatment studies, and avoids the additional uncertainties associated with incorporating non-water components.

## Materials and methods

### Overview

This study was performed using TOPAS-nBio^10^, which provides an interface to Geant4-DNA^11–15^ within TOPAS^16,17^. TOPAS is a toolkit that utilizes most Geant4 libraries^18–20^, thereby enabling applications in radiobiology. TOPAS-nBio version 3.0, implemented based on TOPAS version 4.0, was built upon Geant4 version 11.1.3. Geant4-DNA provides physical processes for interactions of ionizing radiation in the very low-energy region^13,14^, as well as processes for pre-chemical and non-homogeneous chemical interactions^21,22^.

To enable comparison with well-known Auger- and beta-emitting radionuclides, ^64^Cu, ^111^In, ^123^I, ^125^I, ^177^Lu, and ^161^Tb were selected alongside ^134^Ce to evaluate their radiation damage effects. The physical characteristics of each radionuclide are listed in Table 1. Because most AE-emitting radionuclides, including ^134^Ce, also emit CEs, the term “low-energy electrons” is used here to collectively refer to both AEs and CEs. Since low-energy electron-emitting radionuclides also emit other types of radiation, such as gamma rays and X-rays, these radiation types were included in this study, although their effects at the cellular or DNA levels are expected to have a relatively minor contribution.

**Table 1:** Comparison of the radionuclides used in this study in terms of half-life, decay mode, AE and CE yields per decay, and the total AE and CE energy released per decay. The radionuclides considered undergo electron capture (EC) or beta decay (β^−^ and β^+^). More detailed information on the emission spectrum of each radionuclide can be found in^25^.

	Half-life^23^	Decay mode^24^	AE yield per decay^25^	Total AE energy per decay (keV)^25^	CE yield per decay^25^	Total CE energy per decay (keV)^25^
^**134**^ **Ce**	3.16 days	EC : 100%	1.13 E+01	6.67 E+00	1.14 E-02	5.21 E-01
^**64**^ **Cu**	12.70 h	EC & β^+^ : 61.5%; β^−^ : 38.5%	1.81 E+00	2.05 E+00	5.78 E-07	7.73 E-04
^**111**^ **In**	2.80 days	EC : 100%	7.43 E+00	6.88 E+00	1.59 E-01	2.79 E+01
^**123**^ **I**	13.22 h	EC : 100%	1.37 E+01	7.23 E+00	1.59 E-01	2.10 E+01
^**125**^ **I**	59.40 days	EC : 100%	2.30 E+01	1.20 E+01	9.45 E-01	7.28 E+00
^**177**^ **Lu**	6.64 days	β^−^ : 100%	1.27 E+00	1.13 E+00	1.55 E-01	1.35 E+01
^**161**^ **Tb**	6.89 days	β^−^ : 100%	1.10 E+01	8.94 E+00	1.42 E+00	3.93 E+01

## TOPAS-nBio simulation setup

### Physics module

Geant4 is capable of modeling the Auger effect and subsequent Auger cascades that follow ionization, electron capture, or internal conversion by utilizing the Radioactive Decay^26^ and Atomic Relaxation^27,28^ modules. Radioactive decay can be modeled using the G4DecayPhysics and G4RadioactiveDecayPhysics classes, the latter of which is based on the Evaluated Nuclear Structure Data File (ENSDF)^29,30^ library. In TOPAS, the corresponding classes can be implemented as “g4decay” and “g4radioactivedecay”, respectively, by using the built-in GenericIon class with specific atomic number (Z), mass number (A), and ion charge (Q)^31^. This class is compatible with other low-energy electromagnetic physics modules and Geant4-DNA physics models implemented in TOPAS-nBio^31^. The DeexcitationIgnoreCut option was activated to ignore the production cut below a specified threshold. The Fluorescence, Auger, and AugerCascade options were activated to enable the emission of AEs based on the Evaluated Atomic Data Library (EADL) database^32^. The full emission spectrum simulated through these options (including Auger cascades, internal conversion electrons, and associated photons), as well as the resulting secondary particles, were included in the scoring and interpretation.

Ionization and excitation of water molecules were modeled using the electromagnetic modules “g4em-dna” and “TsEmDNAPhysics”, depending on the simulation environment. Although both modules employ the same physics processes and models as G4EmDNAPhysics, the interaction models in TsEmDNAPhysics can be customized by the user. TsEmDNAPhysics integrates multiple Geant4-DNA models for several physical electron interaction processes. These include G4DNABornExcitationModel for excitation (9 eV–1 MeV)^12^, G4DNABornIonisationModel for ionization (11 eV–1 MeV)^12^, G4DNAChampionElasticModel for elastic scattering (7.4 eV–1 MeV)^12^, G4DNAMeltonAttachmentModel for dissociative electron attachment (4 eV–13 eV)^33^, and G4DNASancheExcitationModel for vibrational excitation (2 eV–100 eV)^34^. The physical stage dealing with these interactions are simulated within the first femtosecond. The tracking cut was set to 7.4 eV, the default value determined by the selected G4EmDNAPhysics constructor. More details regarding the interaction processes and the tracking cut are provided elsewhere^14^.

### Chemistry module

As mentioned previously, the chemical processes in TOPAS-nBio consist of a pre-chemical stage followed by a chemical stage. During the pre-chemical stage, which lasts up to 1 ps, several interactions are simulated, including the formation of the initial chemical species produced by the dissociative decay and auto-ionization of ionized or excited water molecules, as well as the thermalization of sub-excitation electrons^35^. The dissociation schemes and branching ratios associated with excited or ionized water molecules must be defined before the generation of the initial chemical species^35^. In the chemical stage, which lasts up to 1 µs, the initial chemical species diffuse and react with each other according to the diffusion coefficients (at a given temperature) and reaction rates, leading to the formation of new species and a decrease in the initial species. Since most of the Geant4-DNA physics processes used here are defined for water, the chemical processes and geometric components were also modeled as water.

The “TsEmDNAChemistry” module^35^ was employed to model the pre-chemical and chemical stages in TOPAS-nBio, and it was developed based on the “G4EmDNAChemistry” constructor. The default branching and dissociation schemes implemented in “TsEmDNAChemistry” during the pre-chemical stage were adopted from the Geant4-DNA chemistry constructor “G4EmDNAChemistry”^21,36^. During the chemical stage, diffusion coefficients and reaction rates were considered. The diffusion coefficients of chemical species in liquid water, as defined in “G4EmDNAChemistry”^37^ and “TsEmDNAChemistry”, are listed in Table 2. The reaction rate constants from “G4EmDNAChemistry”^21,36^, TOPAS-nBio using “TsEmDNAChemistry”^38^, and this study are listed in Table 3.

The TOPASDefaultReaction.txt file, which incorporates reported diffusion coefficient values^38^ and reaction rate constants^39,40^, was used in this study. The values from Geant4-DNA and TOPAS-nBio in Tables 2 and 3 may show slight differences but are generally consistent. The diffusion and reactions of chemical species in chemical scoring were modeled using a step-by-step method^35,41^. ChemicalStageTransportActive was set to true for the chemical species. The transport of chemical species was not simulated over their entire lifetime but was limited to 1 ns, following previous studies^42–44^, with a time-step resolution of 0.5 ps. This limitation indirectly reflects the scavenging conditions of the cellular environment^45^ and improves simulation efficiency.

**Table 2:** Comparison of the diffusion coefficients from Geant4-DNA and this study according to the chemical species; modified from^35^.

Chemical species	Diffusion coefficient (10^− 9^m^2^/s) at 25 °C	
	G4EmDNAChemistry^37^ (from Geant4-DNA)	TsEmDNAChemistry^38^ (in this study)
**Solvated electron (e**_**aq**_^**−**^ **)**	4.9	4.9
**Hydroxyl radical (∙OH)**	2.8	2.2
**Hydrogen (H∙)**	7.0	7.0
**Hydronium (H**_**3**_ **O** ^**+**^ **)**	9.0	9.46
**Dihydrogen (H**_**2**_ **)**	5.0	4.8
**Hydroxide (OH** ^**−**^ **)**	5.0	5.3
**Hydrogen peroxide (H**_**2**_ **O**_**2**_ **)**	1.4	2.3

**Table 3:** Comparison of the reaction rate constants from Geant4-DNA, TOPAS-nBio study in 2018^35^, and this study according to the chemical reactions; modified from^35^.

Chemical reaction	Reaction rate constant k_obs_ (10^10^M^− 1^/s)		
	G4EmDNAChemistry^21,36^ (from Geant4-DNA)	TsEmDNAChemistry^38^ (from TOPAS-nBio (2018))	TsEmDNAChemistry ^39,40^ (in this study)
**e**_**aq**_^**−**^ **+ e**_**aq**_^**−**^ **→ H**_**2**_ **+ 2OH**^**−**^	0.5	0.636	0.55
**e**_**aq**_^**−**^ **+ ∙OH → OH**^**−**^	2.95	2.95	3.0
**e**_**aq**_^**−**^ **+ H∙ → H**_**2**_ **+ OH**^**−**^	2.65	2.5	2.5
**e**_**aq**_^**−**^ **+ H**_**3**_**O**^**+**^ **→ H∙**	2.11	2.11	2.3
**e**_**aq**_^**−**^ **+ H**_**2**_**O**_**2**_ **→ OH**^**−**^ **+ ∙OH**	1.41	1.1	1.1
**∙OH + ∙OH → H**_**2**_ **O**_**2**_	0.44	0.55	0.55
**∙OH + H∙ → H**_**2**_ **O**	1.44	1.55	2.0
**H∙ + H∙ → H**_**2**_	1.2	0.503	0.78
**H**_**3**_**O**^**+**^ **+ OH**^**−**^ **→ H**_**2**_**O**	14.3	11.3	14.3

## Geometry setup and damage scoring

### Dose point kernel

The dose point kernel (DPK) describes the spatial distribution of absorbed dose around a point source as a function of distance. It was evaluated before DNA strand break analysis to characterize the dose contribution of each radionuclide and to provide a basis for subsequent microdosimetric and DNA damage assessments.

The DPKs of each radionuclide were calculated within a water sphere with a radius of 100 μm. Radiation emission was assumed to be isotropic, and the simulations employed the g4em-dna, g4radioactivedecay, and g4decay physics modules.

### DNA model

Radiation damage effects in a single DNA molecule were evaluated using the CharltonDNA model, which assumes that a radionuclide is attached to the DNA. This geometry was adopted to reflect the intracellular behavior of radionuclide-labeled PSMA-617, which undergoes internalization and accumulates in the perinuclear region, potentially placing it in close proximity to DNA strands.

**Fig. 1:**
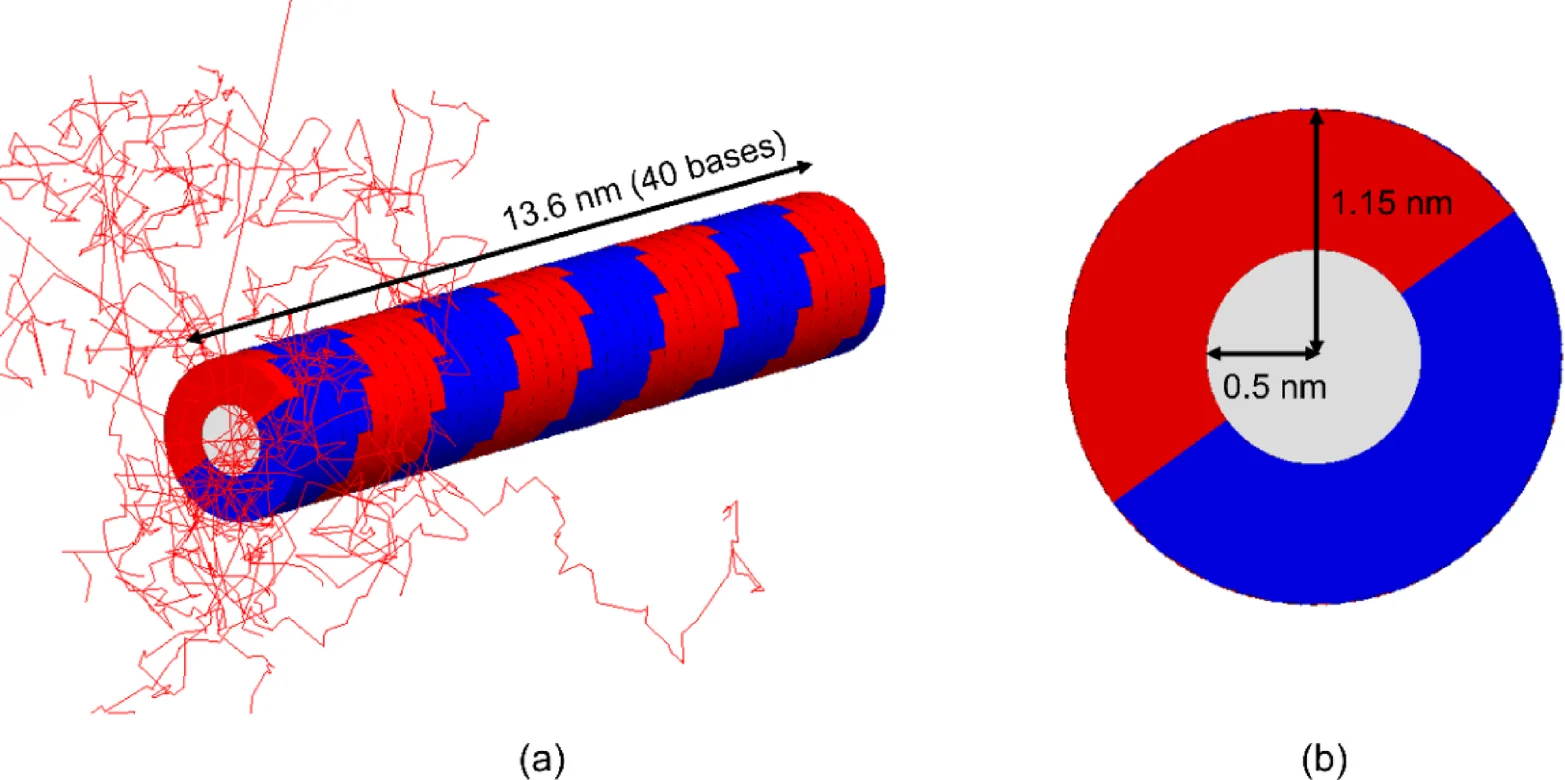
Illustration of the CharltonDNA model with 40 base pairs **(a)** and a cross-section of the CharltonDNA model **(b)** generated by TOPAS-nBio Qt visualization.

The CharltonDNA model is based on^46,47^ and can be implemented using TsCharltonDNA^48^ in TOPAS-nBio. In this model, the central cylinder, with a radius of 0.5 nm and a length of 0.34 nm, represents a single DNA base pair. Two half-cylinders surrounding the central cylinder represent the sugar-phosphate backbone^48^. The backbone is rotated around the central cylinder in 36° increments^49^, resulting in ten base pairs per full helical turn, as shown in Fig. 1. The length of the CharltonDNA model was derived from the simulation geometry described in^49^, which was based on experimental data reported in^50^, and corresponds to a 40-base pair (bp) DNA segment. Each decaying radionuclide was positioned at the central end of the DNA structure without any spatial offset.

### Nucleus model

For more advanced modeling, the TsNucleus model^44^ provided by TOPAS-nBio was used. This model is based on the DNA geometry proposed in^51^ and described in^52^, and represents the entire nucleus of human fibroblasts in the G0/G1 phase using a water-based approach. In this model, the sugar-phosphate backbone volumes are represented as spheres with a radius of 0.271 nm, whereas each base is modeled as an oblate spheroid with a semi-minor axis of 0.185 nm and a semi-major axis of 0.328 nm^53^. The DNA double helix is wrapped around a histone protein, which is represented as a cylinder with a diameter of 6.6 nm and a length of 5.7 nm, forming a nucleosome. Six nucleosomes constitute one turn of the chromatin fiber, and this structure was stacked to form a cylindrical volume of chromatin fiber. The fibers were then distributed into voxels using a 3D Hilbert space-filling curve, and these voxels were repeatedly tiled in a rectangular layout such that a 4.65 μm radius spherical nucleus contained approximately 6 Gbp of DNA. Further details on the concept of this geometry are available in^44^.

### Damage scoring criteria

In TOPAS-nBio, DNA strand breaks are classified as direct or indirect damage. Direct damage results from the deposition of energy directly in the DNA, whereas indirect damage arises when radiolysis products generated by energy deposition interact with the DNA backbone and induce strand breaks. In addition, quasi-direct damage has been described^54^, although its contribution is small. In this process, ionizing radiation can ionize the hydration shell surrounding the DNA, producing holes and electrons that may be transferred to DNA bases or the sugar-phosphate backbone^55^. Because this damage is experimentally indistinguishable from direct ionization, it is treated as a subcategory of direct damage. A DNA hydration shell thickness of 0.16 nm was assumed.

For direct damage, an event was recorded when the energy deposited in the DNA backbone exceeded 17.5 eV^56,57^. This threshold was established by reproducing the results of^58^. The probability of quasi-direct damage originating from the hydration shell was set to 0.33^53,59^. For indirect damage, the probability that a hydroxyl radical binds to the DNA backbone and induces a strand break was set to 0.4, meaning that a hydroxyl radical interacting with the backbone induces damage with a 40% probability. These damage parameters have been adopted in previous studies^44,60^.

A double-strand break (DSB) was recorded when two single-strand breaks (SSBs) occurred on opposite strands within 10 base pairs. DSBs were classified as direct, indirect, or hybrid, the latter arising from a combination of direct and indirect damage. SSBs were classified as either direct or indirect damage. Uncertainty in the results, including damage yield, is reported as the standard error of the mean (SEM)^61^.

The DNADamageStepByStep scorer was used for the CharltonDNA model, whereas the DNADamageNucleusStepByStep scorer was used for the TsNucleus model. Radiation emission was assumed to be isotropic. The simulations employed four modules: TsEmDNAPhysics, TsEmDNAChemistry, g4radioactivedecay, and g4decay. Scavengers such as dimethyl sulfoxide (DMSO) and DNA repair mechanisms were not considered. The decay of ^134^La and its corresponding emission spectrum were not included, as this study focused solely on ^134^Ce.

## Results

### DPK

**Fig. 2:**
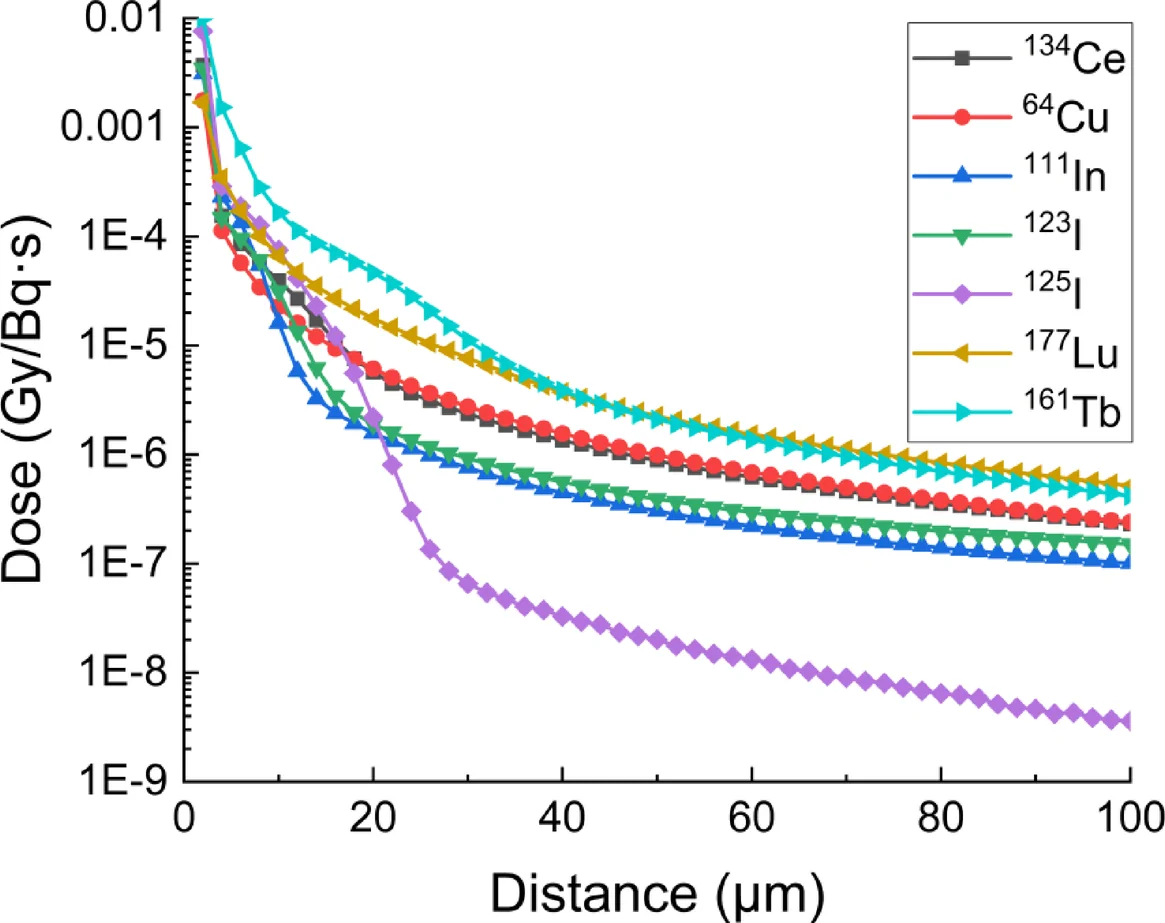
Plot of DPKs of each radionuclide with an interval of 2 μm.

**Fig. 3:**
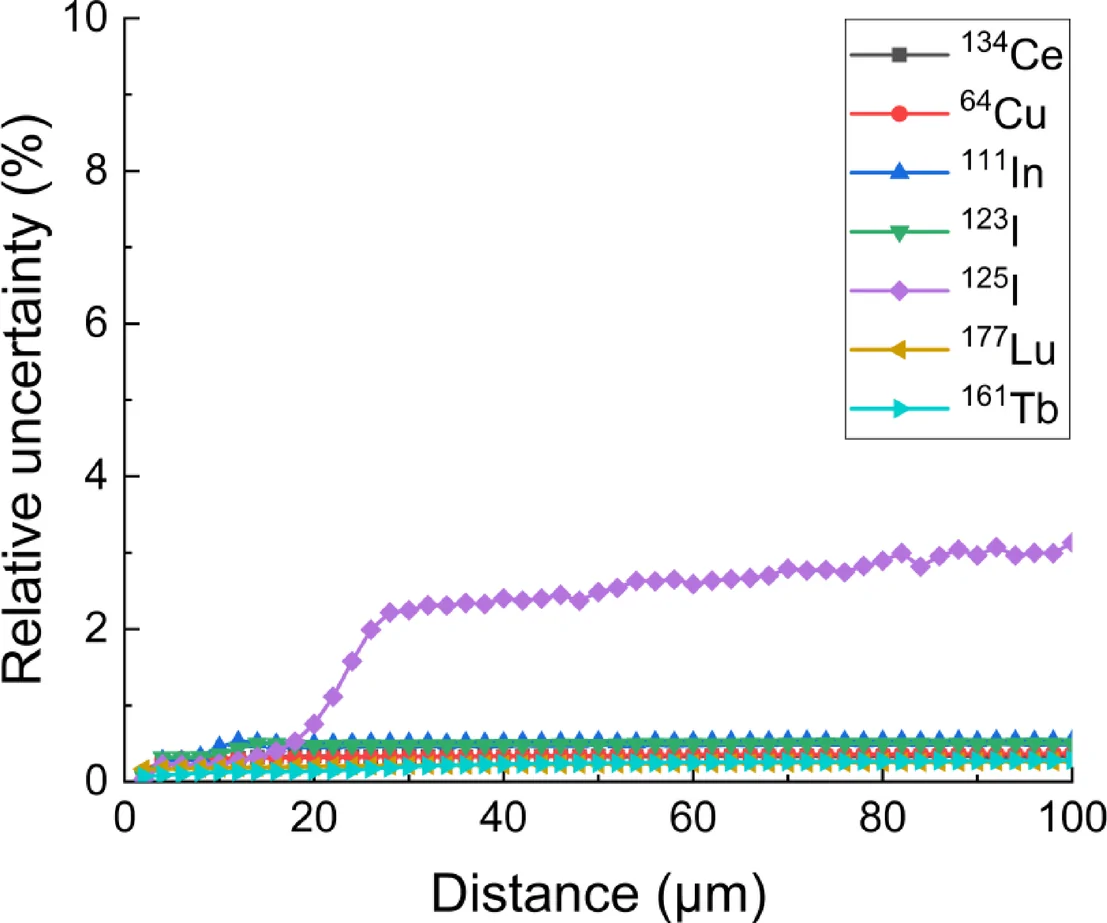
Plot of relative uncertainty of the DPKs for each radionuclide.

Figure 2 shows the DPKs for each radionuclide, calculated using one million decays, on a semi-logarithmic scale, allowing the absorbed dose to be inferred as a function of cell size. As the distance increases, some radionuclides exhibit crossover behavior due to factors such as the number and energy of the low-energy electrons they emit. The relative uncertainties of the DPK results were generally below 10% and are shown in Fig. 3.

### Damage yield in the CharltonDNA model

Table 4 presents the mean DSB yield per decay, and Table 5 presents the mean SSB yield per decay for the case in which a radionuclide was positioned at the center of the CharltonDNA cross-section. For each radionuclide, 10,000 decays were simulated. Because the radionuclide was assumed to be bound to the DNA, direct damage was the dominant contribution, followed by hybrid and indirect damage. Overall, ^161^Tb produced the highest DSB and SSB yields, followed by ^134^Ce, although the difference between them was small. Results from other studies^62–67^ are also included in Tables 4 and 5 for comparison.

**Table 4:** Comparison of per-decay quantities in the CharltonDNA model, including the mean energy imparted and the DSB yields from direct, indirect, and hybrid effects, as well as the total DSB yield for each radionuclide. Total DSB yields per decay reported in other studies are also included for comparison.

	Energy imparted per decay (keV)	DSB direct	DSB indirect	DSB hybrid	DSB total	DSB total in other work
^**134**^ **Ce**	0.5056 ± 0.0024	0.2338 ± 0.0047	0.0532 ± 0.0023	0.1853 ± 0.0041	0.4723 ± 0.0064	–
^**64**^ **Cu**	0.2241 ± 0.0012	0.0670 ± 0.0026	0.0247 ± 0.0016	0.0661 ± 0.0026	0.1578 ± 0.0039	0.171 ± 0.003^62^
^**111**^ **In**	0.4344 ± 0.0024	0.1720 ± 0.0042	0.0480 ± 0.0022	0.1635 ± 0.0040	0.3835 ± 0.0061	1.09 ± 0.01^62^ 0.28^63^ 1.3^64^ 1.39^65^ 1.20^66^
^**123**^ **I**	0.4885 ± 0.0027	0.2338 ± 0.0048	0.0490 ± 0.0022	0.1705 ± 0.0040	0.4553 ± 0.0064	1.20 ± 0.01^62^ 0.56^63^ 0.50^65^ 0.52^66^ 0.747 ± 0.012 ^67^
^**125**^ **I**	0.4285 ± 0.0021	0.1848 ± 0.0042	0.0416 ± 0.0020	0.1548 ± 0.0038	0.3812 ± 0.0059	1.94 ± 0.01^62^ 1.11^63^ 1.70^65^ 1.91^66^ 0.979 ± 0.016^67^
^**177**^ **Lu**	0.4605 ± 0.0029	0.1936 ± 0.0045	0.0506 ± 0.0023	0.1714 ± 0.0041	0.4156 ± 0.0064	–
^**161**^ **Tb**	0.5108 ± 0.0029	0.2283 ± 0.0048	0.0543 ± 0.0024	0.1961 ± 0.0043	0.4787 ± 0.0067	–

**Table 5:** Comparison of per-decay quantities in the CharltonDNA model, including the mean energy imparted and the SSB yields from direct and indirect effects, as well as the total SSB yield for each radionuclide. Total SSB yields per decay reported in other studies are also included for comparison.

	Energy imparted per decay (keV)	SSB direct	SSB indirect	SSB total	SSB total in other work
^**134**^ **Ce**	0.5056 ± 0.0024	0.6795 ± 0.0079	0.4194 ± 0.0063	1.0989 ± 0.0098	–
^**64**^ **Cu**	0.2241 ± 0.0012	0.4340 ± 0.0061	0.3065 ± 0.0054	0.7405 ± 0.0075	–
^**111**^ **In**	0.4344 ± 0.0024	0.6142 ± 0.0078	0.4163 ± 0.0062	1.0305 ± 0.0095	1.35^63^ 2.80^65^ 2.90^66^
^**123**^ **I**	0.4885 ± 0.0027	0.6816 ± 0.0079	0.3909 ± 0.0061	1.0725 ± 0.0096	2.03^63^ 1^65^ 1.06^66^ 2.12 ± 0.02^67^
^**125**^ **I**	0.4285 ± 0.0021	0.6237 ± 0.0077	0.3866 ± 0.0060	1.0103 ± 0.0093	3.45^63^ 3.20^65^ 3.36^66^
^**177**^ **Lu**	0.4605 ± 0.0029	0.6372 ± 0.0079	0.4267 ± 0.0062	1.0639 ± 0.0097	–
^**161**^ **Tb**	0.5108 ± 0.0029	0.6892 ± 0.0083	0.4364 ± 0.0064	1.1256 ± 0.0100	–

### Damage yield in the TsNucleus model

A total of 10,000 decays were simulated to obtain the TsNucleus model results. Table 6 presents the energy imparted per decay and the corresponding absorbed dose for radionuclides randomly distributed within the nucleus but not bound to DNA. Because the TsNucleus model is substantially larger than the CharltonDNA model, the energy deposited per decay in the nucleus was higher. Among the radionuclides, ^125^I exhibited the highest value, followed by ^161^Tb, whereas ^134^Ce was comparable to ^111^In. Overall, the absorbed dose was approximately proportional to the mean energy imparted per decay.

**Table 6:** Average energy imparted per decay and the corresponding absorbed dose in the TsNucleus model for each radionuclide.

	Energy imparted per decay (keV)	Absorbed dose per decay (Gy)
^**134**^ **Ce**	6.3786 ± 0.0409	0.0029 ± 1.85E-05
^**64**^ **Cu**	5.1503 ± 0.0377	0.0023 ± 1.59E-05
^**111**^ **In**	6.3761 ± 0.0583	0.0029 ± 2.41E-05
^**123**^ **I**	6.8715 ± 0.0528	0.0030 ± 2.20E-05
^**125**^ **I**	12.3594 ± 0.0630	0.0049 ± 2.50E-05
^**177**^ **Lu**	5.1626 ± 0.0600	0.0021 ± 2.32E-05
^**161**^ **Tb**	9.8551 ± 0.0840	0.0039 ± 3.23E-05

Table 7 presents the DSB yield per decay for radionuclides in the TsNucleus model, whereas Table 8 presents the corresponding SSB yields per decay. The DSB yields in Table 7 follow trends similar to those of the results in Table 6. Hybrid damage, arising from both direct and indirect effects, accounted for the largest fraction of the total damage, followed by indirect and direct damage.

The SSB yields in Table 8 show a greater contribution from indirect damage than from direct damage, indicating a more prominent role of indirect effects under these conditions. When normalized to dose, the results presented in Table 9 differ from the trends observed for per-decay damage yields, highlighting the importance of considering damage yield efficiency. Results from other studies^68,69^ are also included in Tables 7 and 8 for comparison.

**Table 7:** Comparison of per-decay DSB yields arising from direct, indirect, and hybrid effects, along with the total DSB yield in the TsNucleus model for each radionuclide. Total DSB yields per decay reported in other studies are also included for comparison.

	DSB direct	DSB indirect	DSB hybrid	DSB total	DSB total in other work
^**134**^ **Ce**	0.0178 ± 0.0014	0.0273 ± 0.0017	0.0436 ± 0.0021	0.0887 ± 0.0032	–
^**64**^ **Cu**	0.0085 ± 0.0009	0.0196 ± 0.0014	0.0268 ± 0.0017	0.0549 ± 0.0024	0.187 ± 0.001^68^
^**111**^ **In**	0.0169 ± 0.0013	0.0289 ± 0.0017	0.0445 ± 0.0022	0.0903 ± 0.0031	≈ 0.561^68^
^**123**^ **I**	0.0168 ± 0.0013	0.0306 ± 0.0018	0.0474 ± 0.0022	0.0948 ± 0.0033	–
^**125**^ **I**	0.0246 ± 0.0016	0.0454 ± 0.0022	0.0712 ± 0.0027	0.1412 ± 0.0040	≈ 0.748^68^
^**177**^ **Lu**	0.0091 ± 0.0009	0.0169 ± 0.0013	0.0267 ± 0.0017	0.0527 ± 0.0024	8.98 × 10^− 3^^69^
^**161**^ **Tb**	0.0174 ± 0.0013	0.0338 ± 0.0019	0.0468 ± 0.0022	0.0980 ± 0.0033	9.19 × 10^− 3^^69^

**Table 8:** Comparison of per-decay SSB yields arising from direct and indirect effects, along with the total SSB yield in the TsNucleus model for each radionuclide. Total SSB yields per decay reported in other studies are also included for comparison.

	SSB direct	SSB indirect	SSB total	SSB total in other work
^**134**^ **Ce**	0.4984 ± 0.0081	1.2714 ± 0.0160	1.7698 ± 0.0203	–
^**64**^ **Cu**	0.3821 ± 0.0070	1.0674 ± 0.0138	1.4495 ± 0.0172	–
^**111**^ **In**	0.4931 ± 0.0084	1.3466 ± 0.0179	1.8397 ± 0.0226	–
^**123**^ **I**	0.5117 ± 0.0085	1.3698 ± 0.0173	1.8815 ± 0.0220	–
^**125**^ **I**	0.7958 ± 0.0106	2.2834 ± 0.0225	3.0792 ± 0.0284	–
^**177**^ **Lu**	0.4284 ± 0.0078	1.0825 ± 0.0164	1.5109 ± 0.0209	1.04 × 10^− 1^^69^
^**161**^ **Tb**	0.6860 ± 0.0105	1.9626 ± 0.0232	2.6486 ± 0.0297	1.08 × 10^− 1^^69^

**Table 9:** Comparison of DSB/Gy/Gbp, SSB/Gy/Gbp, and the SSB-to-DSB ratio in the TsNucleus model for each radionuclide.

	DSB/Gy/Gbp	SSB/Gy/Gbp	SSB-to-DSB ratio
^**134**^ **Ce**	6.2342 ± 0.2876	127.6999 ± 3.1061	20.4839
^**64**^ **Cu**	4.7834 ± 0.4399	149.5673 ± 3.3105	31.2679
^**111**^ **In**	6.8279 ± 0.3520	129.5590 ± 2.2519	18.9749
^**123**^ **I**	6.6755 ± 0.3715	124.3985 ± 2.2019	18.6352
^**125**^ **I**	5.0245 ± 0.1716	107.6319 ± 0.9698	21.4214
^**177**^ **Lu**	3.8599 ± 0.3032	221.4332 ± 9.1200	57.3671
^**161**^ **Tb**	4.1910 ± 0.2647	174.1577 ± 8.0860	41.5553

## Discussion

This study compared the DPKs of ^134^Ce with those of other radionuclides within a water sphere and evaluated radiation damage yields using the CharltonDNA and TsNucleus models in TOPAS-nBio. In both models, scorers specifically designed to record DNA damage were employed to provide a more realistic representation of damage outcomes than the DBSCAN algorithm^70^, which has been commonly used in previous studies.

The DPK results showed that ^134^Ce and other radionuclides (excluding ^177^Lu and ^161^Tb) consistently produced lower absorbed doses over the relevant distance range than the beta-emitting radionuclides ^177^Lu and ^161^Tb. This trend can be attributed to low-energy electrons, which lead to more localized energy deposition over shorter distances. These results suggest that close proximity of these radionuclides to DNA is advantageous, thereby supporting the use of the geometries considered in this study. The DPK results for ^177^Lu and ^161^Tb^71^, as well as for ^111^In, ^123^I, and ^125^I^72,73^ can be referred from previous studies. Although not shown in the figures, the photon contribution to the dose did not exceed 0.92% (^134^Ce), 0.79% (^64^Cu), 2.80% (^111^In), 1.28% (^123^I), 1.00% (^125^I), 0.13% (^177^Lu), and 0.24% (^161^Tb) within 10 μm in the DPK results, indicating that photon contributions are minor at the geometric scales considered in this study.

The CharltonDNA geometry was used to quantify and compare the intrinsic energy deposition and damage yield patterns within a single DNA molecule arising from physical differences among radionuclides. ^134^Ce exhibited an effect that was slightly lower than, but still highly comparable to, that of ¹⁶¹Tb, which has recently received considerable attention. For total DSBs, ^134^Ce achieved 98.66% of the performance of ^161^Tb, whereas for total SSBs, it achieved 97.63%. The DSB results obtained in this geometry are generally proportional to the energy imparted to the DNA and are comparable across radionuclides, except for ^64^Cu, which shows a lower effect because of its relatively weaker low-energy electron component. Overall, these findings suggest that the radiation damage effect from ^134^Ce decay at the single DNA level is comparable to that of widely used radionuclides.

In the TsNucleus model, when radionuclides are randomly distributed within the nucleus, the distance between the radionuclides and DNA increases, leading to a reduced contribution of direct damage. Instead, the contribution from chemical species that diffuse to the DNA increases, resulting in a higher proportion of indirect and hybrid damage. ^125^I exhibited the highest performance for both DSBs and SSBs per decay, followed by ^161^Tb. Compared to ^125^I, ^134^Ce showed 62.82% and 57.48% effectiveness for the total DSBs and SSBs, respectively. Compared with ^161^Tb, ^134^Ce demonstrated 90.51% and 66.82% effectiveness for the total DSBs and SSBs, respectively. Although mechanisms such as the crossfire effect are not considered in this geometry, they will be investigated in future studies.

Among the factors that may have contributed to the differences between the results of this study and others (Tables 4, 5 and 7, and 8), the adopted geometry and the positions of radionuclides within those geometries likely played a critical role. Other contributing factors include the choice of physics and chemistry models, the use of average or full emission spectra, and the energy and time thresholds associated with direct and indirect damage. In terms of direct damage, linear probability from 5 eV to 37.5 eV^74^ could also be used instead of the 17.5 eV threshold, and this option was reported to increase both SSB and DSB^43,60,75–77^. Using fixed thresholds other than 17.5 eV also results in different outcomes^60^. A change in the chemical stage duration also affects the results, as it has been shown to increase the number of DSBs when extended from 1 ns to 10 ns^76^.

Returning to the TsNucleus results, the total energy imparted and absorbed dose per decay by ^125^I within the TsNucleus model are higher than those of other radionuclides since ^125^I emits the greatest number of AEs and the highest total AE energy, along with the second-highest number of CEs and total CE energy. This contributes to higher strand break yields per decay. Meanwhile, these results should not be attributed solely to AEs. Given that the energy of AEs is generally small enough to be deposited within the nanometer scale, CEs may play a more decisive role in a micrometer-scale nucleus than AEs due to their longer range, as suggested by previous studies comparing ^177^Lu and ^161^Tb^78,79^. The respective contributions require further investigation.

^125^I and ^161^Tb exhibited lower DSB/Gy/Gbp values than the other radionuclides, indicating lower dose-normalized damage efficiency in the nucleus geometry. This discrepancy, compared with the DNA damage yield per decay, arises from the higher local absorbed dose. Under such conditions, an increase in absorbed dose does not translate proportionally into an increase in DSB yield, resulting in reduced dose-normalized efficiency. In contrast, radionuclides such as ^134^Ce, ^111^In, and ^123^I showed higher DSB/Gy/Gbp values, indicating more efficient DSB production per unit absorbed dose, despite producing fewer absolute DSBs per decay. Since ^125^I and ^161^Tb have half-lives more than twice that of ^134^Ce, consideration of the injected activity suggests that they may deliver excessively high doses despite their lower radiation damage efficiency. Therefore, analysis of ^134^Ce considering multiple factors, such as damage efficiency and half-life, requires further and more comprehensive studies.

Despite these findings, several limitations should be noted. DNA repair and scavenging effects were not considered, and only the hydroxyl radical was modeled as the primary chemical species responsible for indirect damage; these factors may have influenced the DNA damage results. In addition, assuming most of the environment (e.g., DNA composition and chromatin structure) as liquid water does not fully reflect the actual energy deposition^80,81^ and this would affect the outcomes of DNA damage. The close proximity between the radionuclide and DNA geometry may not realistically represent the biological environment. It should also be noted that the use of different physics/chemistry models or material cross-sections than those employed in this study could lead to different results, which constitutes one of the uncertainties associated with this type of modeling approach. Most importantly, therapeutic efficacy cannot be fully characterized by numerical metrics such as absorbed dose or strand break yields alone, but requires evaluation of biological outcomes (e.g., cell killing and tumor control) supported by experimental validation.

Nevertheless, this study is significant because it investigated the DNA damage yields of ^134^Ce at both the DNA and nucleus levels using Monte Carlo simulation. The observed DNA damage results of ^134^Ce is comparable to conventional radionuclides, indicating the potential for further research. In addition, both physical and chemical processes were incorporated to provide a more realistic representation of the underlying mechanisms and to support the results. If contributions from ^134^La decay are included, the DNA damage yield would likely be greater than that reported for ^134^Ce alone in this study. Future studies will aim to compare treatment outcomes by incorporating factors such as DNA repair and dose rate, which may vary among radionuclides, together with experimental approaches such as the comet assay. Further extensions could include the investigation of lineal energy characteristics^82^ to provide additional microdosimetric insight into radiation quality effects. Although previous studies have often relied on monoenergetic assumptions or discrete energy distributions, incorporating the full emission spectrum in future analyses may further improve radiobiological interpretation.

## Conclusion

This study examined the radiation damage effects of ^134^Ce, along with radionuclides that undergo EC and beta decay, from both dosimetric and DNA-scale perspectives. The radionuclides considered could theoretically be used for theranostic applications; however, most emit gamma rays and require single-photon emission computed tomography (SPECT), which offers lower spatial resolution than PET. Even when PET imaging is feasible with certain radionuclides, the associated DNA damage outcomes may be relatively low. Although the diagnostic aspect was not addressed in this study, PET imaging is enabled by the decay of ^134^La to ^134^Ba following ^134^Ce decay. If the DNA damage effects of ^134^Ce identified in this study are also taken into account, the use of this radionuclide could be further justified.

## Data Availability

The data supporting this study will be made available upon reasonable request.
